# Distinct copy number signatures between residual benign and transformed areas of carcinoma ex pleomorphic adenoma

**DOI:** 10.1038/s41598-024-63763-9

**Published:** 2024-10-09

**Authors:** João Figueira Scarini, Wellington Lima Sabino, Reydson Alcides de Lima-Souza, Erika Said Abu Egal, Alfio José Tincani, Rogério Gondak, Luiz Paulo Kowalski, Ana Cristina Victorino Krepischi, Albina Altemani, Fernanda Viviane Mariano

**Affiliations:** 1 Department of Pathology, Faculty of Medical Sciences (FCM), University of Campinas (UNICAMP), 126, Tessalia Vieira de Camargo Street, Cidade Universitaria, Campinas, São Paulo 13083887 Brazil; 2 Department of Oral Diagnosis, Piracicaba Dental School (FOP), University of Campinas (UNICAMP), Piracicaba, São Paulo Brazil; 3 Department of Pathology, School of Medicine, University of Utah (UU), Salt Lake City, UT USA; 4 Department of Head and Neck Surgery, Faculty of Medical Sciences (FCM), University of Campinas (UNICAMP), Campinas, São Paulo Brazil; 5 Department of Pathology, Federal University of Santa Catarina (UFSC), Florianópolis, Santa Catarina Brazil; 6 Department of Head and Neck Surgery and Otorhinolaryngology, A.C. Camargo Cancer Center, São Paulo, São Paulo Brazil; 7 Department of Genetics and Evolutionary Biology, Institute of Biosciences, University of São Paulo (USP), São Paulo, São Paulo Brazil

**Keywords:** Array-based comparative genomic hybridization, Copy number alteration, Gene, Carcinoma ex pleomorphic adenoma, Pleomorphic adenoma, Cancer, Cancer genetics, Cancer genomics, Head and neck cancer, Tumour biomarkers, Tumour heterogeneity, Cancer

## Abstract

The mechanisms involved with the pathogenesis of carcinoma ex pleomorphic adenoma (CXPA) seem to be associated with the accumulation of molecular alterations in the pleomorphic adenoma (PA). In this sense, using array-based comparative genomic hybridization (aCGH) a rare series of 27 cases of CXPA and 14 residual PA (rPA) adjacent to the transformation area, we investigated the profile of the copy number alterations (CNAs) comparing benign residual and transformed areas. The main findings were correlated with the histopathological classification by histologic subtype and degree of invasion. The distribution of losses (*p* = 0.187) and amplifications (*p* = 0.172) was not statistically different between rPA and CXPA. The number of gains was increased in the transformed areas compared to the benign residual areas (*p* = 0.005). *PLAG1* gain was maintained along the malignant transformation, as it was observed in both residual PA and CXPA samples, likely being an earlier event during transformation. The amplification of *GRB7* and *ERBB2* may also be an initial step in the malignant transformation of PA to CXPA (salivary duct carcinoma subtype). Furthermore, the amplification of *HMGA2* and *RPSAP52* were the most prevalent alterations among the studied samples. It was noteworthy that amplified genes in the transformed areas of the tumors were enriched for biological processes related to immune signaling. In conclusion, our results underscored for the first-time crucial CNAs in CXPA, some of them shared with the residual benign area adjacent to the transformation site. These CNAs included *PLAG1* gain, as well as amplification of *GRB7*, *ERBB2*, *HMGA2*, and *RPSAP52*.

## Introduction

Carcinoma ex pleomorphic adenoma (CXPA), a tumor resulting from the malignant transformation of pleomorphic adenoma (PA), is a rare and aggressive tumor. Its pathogenesis has attracted considerable research interest over the past two decades^1–8^. By definition, CXPA must show histologic evidence of coexisting residual benign areas (residual PA-rPA) or pre-existing tumor (prior histologic diagnosis of PA in the patient’s medical history). The diagnosis of CXPA is not self-sufficient and should include the carcinomatous phenotype developed in the transformed carcinoma and the extent of capsule invasion of the rPA^9^.

Regarding the malignant area, carcinomas composed of luminal cells only are more likely to develop within a PA, typically adenocarcinoma not otherwise specified (AdNOS) or salivary duct carcinoma (SDC). CXPA with a myoepithelial component has also been reported, notably myoepithelial carcinoma (MC) and epithelial–myoepithelial carcinoma (EMEC)^2,3^. Other less common subtypes include squamous cell carcinoma (SCC), sarcomatoid carcinoma (SC), mucoepidermoid carcinoma (MEC), and adenoid cystic carcinoma (AdCC)^10^. In terms of invasion, the most widely accepted classification defines intracapsular CXPA (iCXPA) as a tumor with neoplastic cells confined to the capsule, minimally invasive CXPA (mCXPA) as tumor extension up to 1.5 mm into extracapsular tissues, and frankly invasive CXPA (fCXPA) as a tumor with extracapsular invasion greater than 1.5 mm^10^.

Taken together, the mechanisms involved in the pathogenesis of CXPA, regardless of histologic subtype or degree of invasion, appear to be related to the accumulation of molecular alterations in the PA. PAs are characterized by recurrent genetic alterations, particularly *PLAG1* and *HMGA2* translocations, which are widely recognized as genetic hallmarks of these tumors^11^. In CXPA, loss of heterozygosity in the 12q region and additional alterations in 17q^12^, deletions of 5q23.2–q31.2, gains of *PLAG1* and *MYC*, and amplifications of *MDM2*, *ERBB2*^13^, and *HMGA2*^14^ are frequently reported CNAs.

With the advent of array-based comparative genomic hybridization (aCGH), our group has previously investigated the copy number alterations (CNAs) profile of PA carcinogenesis^15^ and recurrent PA, with its impact on malignant transformation^16^, the correlation of the genetic profile of recurrent and nonrecurrent PA^17^, and more recently, the role of CNAs encompassing miRNAs in the CXPA development^18^. However, a comparative study of gains, losses, and amplifications in the benign residual and transformed areas of CXPA is lacking.

Therefore, in this study, we applied aCGH to analyze a rare series of 27 CXPA samples aiming to identify differences between their CNAs profile of benign residual and transformed areas. In addition, we correlated the main CNAs findings with histopathologic classification according to histologic subtype and degree of invasion.

## Results

### Clinical, microscopic and genomic profile of analyzed tumors

In this cohort, there was no predilection between males (13 out of 27–48.1%) and females (10 out of 27–37%). The mean age was 58.1 years (range 27–82, ± 13.8). The majority of cases involved the parotid gland (22 out of 27–81.5%), followed by the submandibular gland (2 out of 27–7.4%) and minor salivary glands (2 out of 27–7.4%). Sex information was not available for four patients (14.8%) and site information was not available for one patient (3.7%).

**Figure 1:**
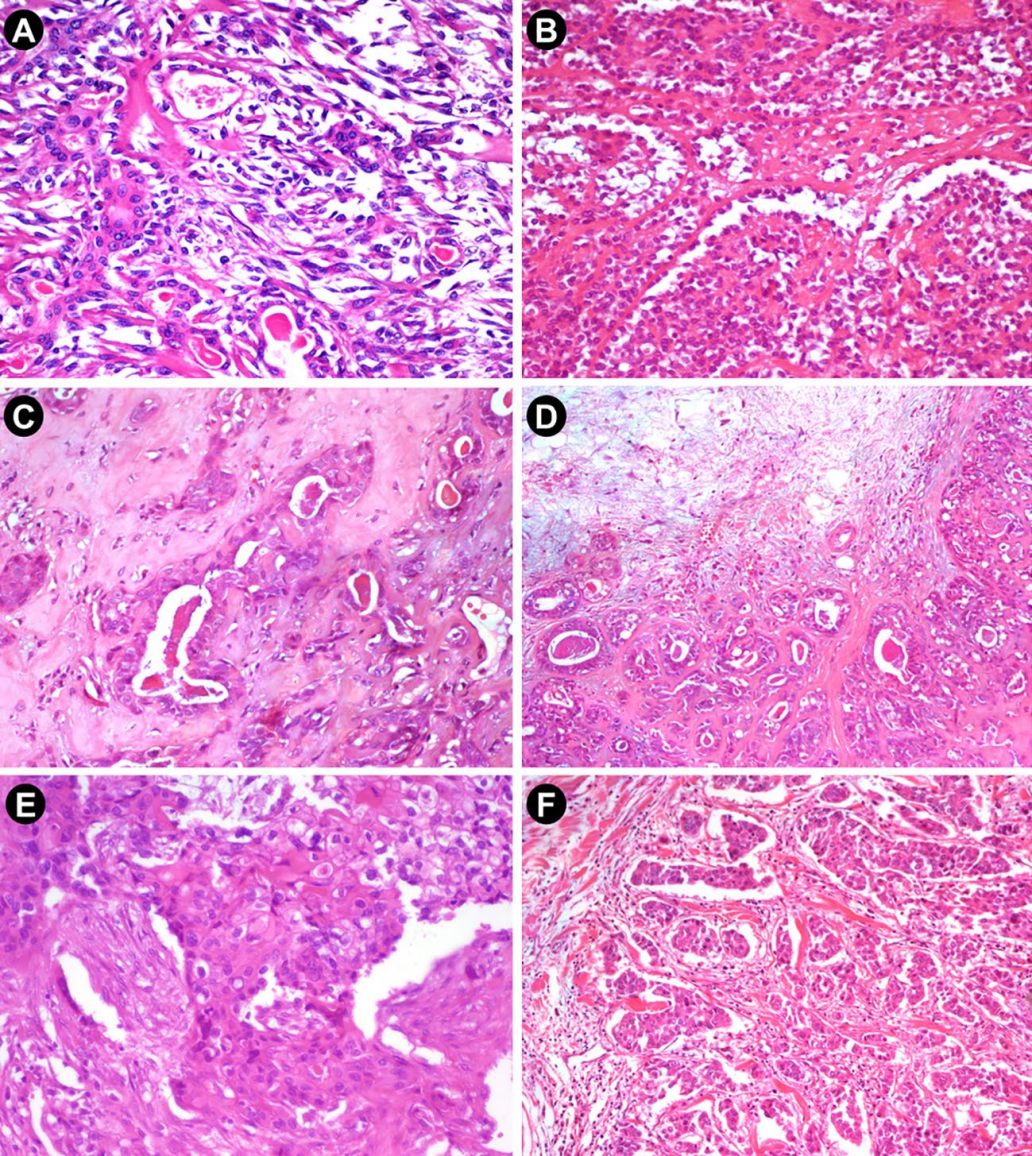
Representative illustration of the major histopathologic subtypes of CXPA used in this study. (**A**) EMEC ex-PA: Proliferation of transformed epithelial and myoepithelial cells within a myxoid stroma (H&E, × 20). (**B**) Growth pattern of MC ex-PA, with cells showing cellular pleomorphism (H&E, × 20). (**C**) AdNOS ex-PA: Tissue fragment shows cells with intense cellular pleomorphism in an invasive pattern, lacking features of other specific types. In situ component present (H&E, × 20). (**D**) Adenocarcinoma with residual benign area (rPA): lower magnification shows coexistence of residual benign and transformed areas (H&E, × 10). (**E**) SCC ex-PA: Cells with epithelioid appearance, and marked cellular pleomorphism, indicating squamous nature of the tumor (H&E, × 20). (**F**) SDC ex-PA: cells with abundant eosinophilic cytoplasm forming cords, nests, and mild cribriform structures within a desmoplastic stroma. In situ component present (H&E, × 10). *PA* pleomorphic adenoma, *CXPA* carcinoma ex pleomorphic adenoma, *rPA* residual pleomorphic adenoma, *EMEC* epithelial-myoepithelial carcinoma, *MC* myoepithelial carcinoma, *AdNOS *adenocarcinoma not otherwise specified, *SCC* squamous cell carcinoma, *SDC* salivary duct carcinoma.

The microscopic profile of the samples analyzed in this study is summarized in Table 1 and Fig. 1. Of the 27 CXPA cases analyzed, 13 (48.1%) carry losses in known cancer genes, whereas this was observed in eight (57.1%) of the 14 rPA cases analyzed.

**Table 1:** Microscopic profile of the 27 analyzed tumors.

Microscopic features	N (%)
Histologic subtype	
AdNOS	6 (22.2)
SCC	1 (3.7)
EMEC	4 (14.8)
MC	6 (22.2)
SDC	9 (33.3)
SC	1 (3.7)
Degree of invasion	
iCXPA	5 (18.5)
mCXPA	5 (18.5)
fCXPA	17 (62.9)
rPA	
Present	14 (51.8)
Absent	13 (48.2)

*CXPA* carcinoma ex pleomorphic adenoma, *rPA* residual pleomorphic adenoma, *AdNOS* adenocarcinoma not otherwise specified, *SCC* squamous cell carcinoma, *EMEC* epithelial–myoepithelial carcinoma, *MC* myoepithelial carcinoma, *SDC* salivary duct carcinoma, *SC* sarcomatoid carcinoma, *iCXPA* intracapsular CXPA, *mCXPA* minimally invasive CXPA, *fCXPA* frankly invasive CXPA.

**Figure 2:**
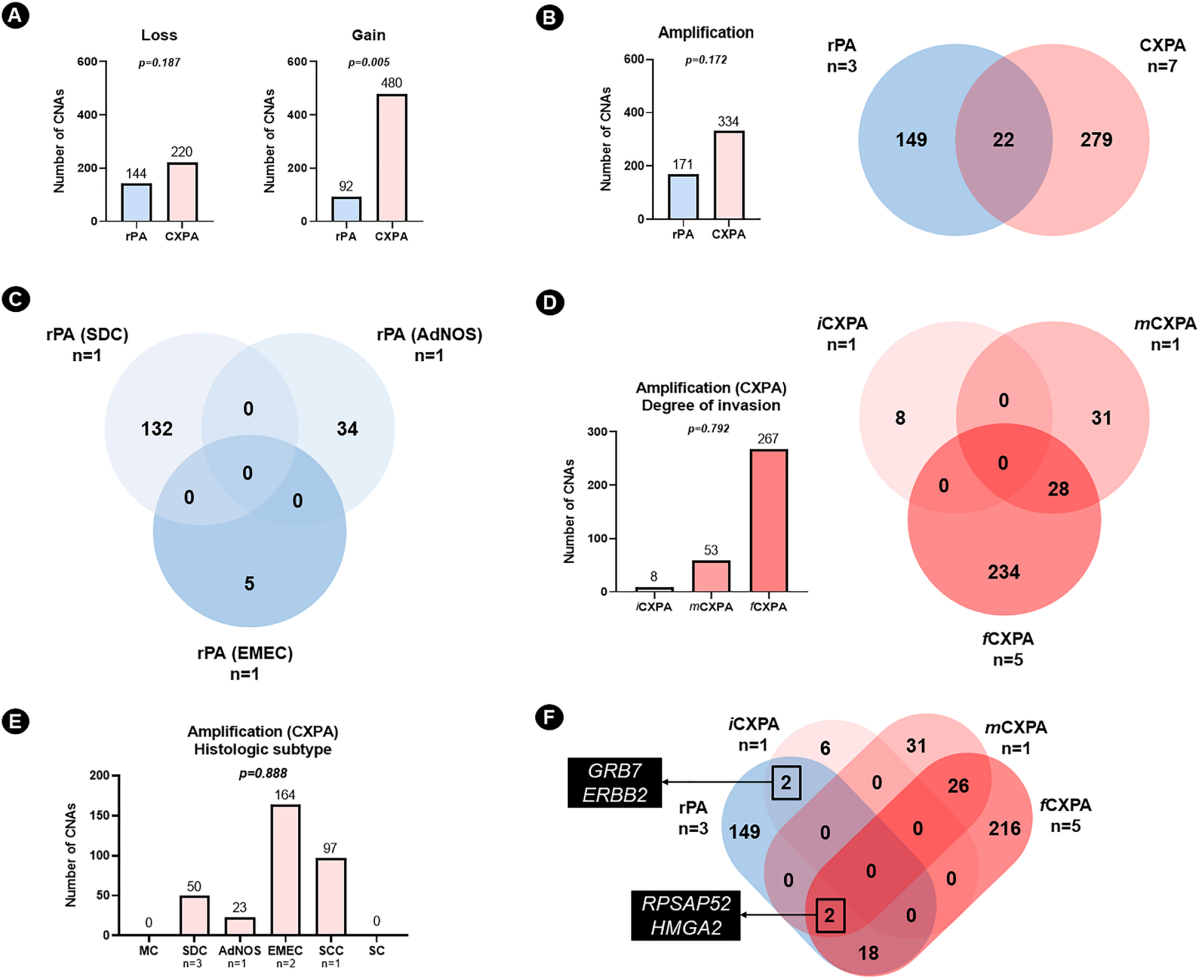
Profile of losses, gains and amplifications between the benign residual area and the transformed area of CXPA included in this study. (**A**) Profile of gains and losses between the benign residual area (rPA) and the transformed area of carcinoma ex pleomorphic adenoma (CXPA) cases included in the study. Note that the number of gains (*p* = 0.005) increases in the transformed area compared to the benign area. The transformed areas showed a tendency towards gene loss compared to the benign residual areas (*p* = 0.187). Mann–Whitney U Test. (**B**) The transformed areas showed a tendency towards gene amplifications compared to the benign residual areas (*p* = 0.172); Twenty-two of them were common to both groups. Note that the Venn diagram excludes repetitions of copy number alterations (CNAs). *Mann–Whitney U Test*. (**C**) Amplification of rPAs was unique to each subgroup. (**D**) There was no statistically significant difference in the number of amplifications per histological subtype of CXPA (*p* = 0.792); some amplifications were shared between minimally invasive CXPA (mCXPA) and frankly invasive CXPA (fCXPA). No amplifications of iCXPA were shared with mCXPA and fCXPA. Kruskal–Wallis Test*.* (**E**) Among all histologic subtypes presented, more amplifications were observed in the two cases of CXPA (EMEC subtype) with identified CNAs. However, no statistically significant difference was found among the analyzed subtypes (*p* = 0.888). Kruskal–Wallis Test*.* (**F**) To better understand the 22 shared genes between rPA and CXPA, we plotted a graph dividing the CXPA group according to the degree of invasion. Amplification of the *GRB7* and *ERBB2* genes was observed between rPA and iCXPA. *RPSAP52* and *HMGA2* amplification were shared between rPA and both mCXPA and fCXPA. *CXPA* carcinoma ex pleomorphic adenoma, *rPA* residual pleomorphic adenoma, *AdNOS* adenocarcinoma NOS, *SCC* squamous cell carcinoma, *EMEC* epithelial–myoepithelial carcinoma, *MC* myoepithelial carcinoma, *SDC* salivary duct carcinoma, *SC* sarcomatoid carcinoma, *iCXPA* intracapsular CXPA, *mCXPA* minimally invasive CXPA, *fCXPA* frankly invasive CXPA, *n* number of samples included in the analysis.

The transformed areas (220 losses—60.4%) showed a tendency towards gene loss compared to the benign residual areas (144 losses—39.6%) (*p* = 0.187). Regarding gains, 15 (55.6%) CXPA carry losses in know cancer genes, whereas this was observed in six (42.9%) rPA cases. Gene gains were more frequent in the transformed areas (480 gains—83.9%) than in the benign residual areas (92 gains—26%) (*p* = 0.005) (Supplementary files 1 and Supplementary file 2) (Fig. 2a). Of the 27 CXPA cases analyzed, seven (25.9%) showed amplifications, whereas this was observed in only three (21.4%) of the 14 rPA cases analyzed. We identified 505 amplifications in the examined tissues (Supplementary file 1). The transformed areas showed a tendency towards gene amplifications (334 amplifications—66.1%) compared to the benign residual areas (171 amplifications—33.9%) (*p* = 0.172) (Fig. 2b). In the Venn diagram, repeated CNAs are not considered, so the lower total number of CNAs in the CXPA reflects more repetitions of CNAs in that group.

### Gains and losses in rPA and CXPA

The majority of samples analyzed showed a profile of frequent gains and losses in known cancer-related genes (Supplementary file 1). In the benign areas, losses were more frequent on chromosomes 17q (4 cases—28.6%), 5q and 6q (three cases each—21.4%) (Supplementary file 2). Among all identified losses, those affecting the genes *CD74*, *EBF1*, *ITK*, *NPM1*, *NSD1*, *PDGFRB*, *RANBP17*, *STL*, *TLX3*, and *TNFAIP3* were more frequently (three losses of 144 each—2.1%). Conversely, gains on 8p and 8q (3 cases each—21.4%) were most frequently observed (Supplementary file 2). The most frequent gains observed involved the genes *PLAG1* and *TCEA1* (four gains of 92 each—4.3%), *HOOK3* (three gains of 92—3.3%), and *CHCHD7* (two gains of 92—2.2%).

In contrast, losses on chromosomes 8p (five cases—18.5%), 2q (four cases—14.8%), and 5q (three cases—11.1%) were most frequently observed in the transformed areas (Supplementary file 2). Notable losses included *WRN, PCM1* (five losses of 220 each—2.3%)*, BCL2*, *CD74*, *ECT2L*, *EZR*, *FGFR1OP*, *GOPC*, *MALT1*, *MLLT4*, *MYB*, *PDGFRB*, *PRDM1*, *ROS1*, *STL*, *TNFAIP3*, and *WHSC1L1* (three losses of 220 each—1.4%). On the other hand, gains on 6p, 8p (four cases each—14.8%), 3q, 8q, 17q, and 22q (three cases each—11.1%) were more frequent (Supplementary file 2). Gene gains more frequently involving the genes *TCEA1* and *PLAG1* (seven gains of 480 each—1.5%) although gains of *CHCHD7*, *MLF1* (six gains of 480 each—1.3%), *BCL3*, *COX6C*, *HEY1*, *HOOK3*, and *NCOA2* (five gains of 480 each—1%) were also observed.

We grouped all cytobands with CNAs. Interestingly, we observed gains on chromosome 8p11.22q12.1 in two rPA cases (samples 1A7 and 3B1), involving the genes *PLAG1*, *HOOK3*, and *TCEA1*. Gains on chromosome 22q11.1–q13. 33 were observed in two cases: fCXPA (SDC subtype) and fCXPA (EMEC subtype), involving the genes *BCR*, *CHEK2*, *CLTCL1*, *EP300*, *EWSR1*, *MKL1*, *MN1*, *MYH9*, *NF2*, *PDGFB*, and *SMARCB1*. Loss on chromosome 8p23.3p11.1 was observed in an iCXPA (SDC subtype) and in a fCXPA (SCC subtype) with alterations in the genes *FGFR1*, *HOOK3*, *PCM1*, *WHSC1L1*, and *WRN*. Finally, a loss at 8p23.3-p11.23 was detected in an iCXPA (SDC subtype) and in a fCXPA (SDC subtype), involving the genes *PCM1, WHSC1L1,* and *WRN*.

### Amplification in rPA

Among the 505 identified amplifications, 149 (29.5%) were exclusively detected in the benign residual area, whereas 22 (4.4%) were shared with the transformed area (Fig. 2b). Amplification was observed in rPAs adjacent to only three histologic subtypes of CXPA: SDC (132 of 171, 77.2%), AdNOS (34 of 171, 19.9%), and EMEC (5 of 171, 2.9%). However, there was no statistically significant difference in the number of amplifications per histologic subtype (*p* = 0.888) (Supplementary file 3) (Fig. 2e). Interestingly, all amplifications identified were exclusive to each subgroup. We performed a search for recurrent amplifications among the analyzed rPA samples, but each amplification appeared only once in these tissues.

### Amplification in CXPA

Among all the amplifications not recurrently detected in CXPA (unique events), 279 (55.2%) were exclusive to the transformed areas (Fig. 2b). When analyzing these tumors based on their degree of invasion, there was a tendency for the number of amplified genes to increase as the degree of invasion and aggressiveness increased (*p* = 0.792): 8 genes were associated with iCXPA (2.4%), 59 with mCXPA (17.7%), and 267 with fCXPA (79.9%). No common amplification was detected between iCXPA and the other two groups. Comparing mCXPA and fCXPA, 28 amplified genes were shared between these groups: *BEST3*, *CCT2*, *CNOT2*, *CPM*, *CPSF6*, *FRS2*, *HMGA2*, *KCNMB4*, *LOC100507195*, *LOC100507250*, *LOC101928002*, *LOC101928062*, *LRRC10*, *LYZ*, *MDM1*, *MDM2*, *MIR1279*, *MIR3913-1*, *MIR3913-2*, *MIR6074*, *NUP107*, *PTPRB*, *RAB3IP*, *RAP1B*, *RPSAP52*, *SLC35E3*, *SNORA70G*, and *YEATS4* (Fig. 2d).

Regarding the histologic subtype of these tumors, only CXPA classified as MC subtype and CXPA classified as SC subtype did not present amplifications. CXPAs with the EMEC subtype had more genes amplified (164–49.1%) than the SCC subtype (97–29%), the SDC subtype (50–15%) and the AdNOS subtype (23–6.9%). However, there was no statistically significant difference in the number of amplifications per histologic subtype (*p* = 0.134) (Supplementary file 3) (Fig. 2e).

As for the rPA, we looked for recurrent amplifications among the analyzed CXPA samples (Supplementary file 3). Amplifications of *HMGA2* (3 amplifications—1%) and *RPSAP52* (3 amplifications—1%) were more frequent. Another 36 amplifications were detected twice when we analyzed all malignant areas that showed alteration: *AGAP2-AS1*, *BVES-AS1, CCT2, CDK12*, *CDKN2B-AS1*, *CNTN5*, *COM, CPSF6, ERBB2*, *FGFR1*, *FRS2*, *GRB7, IFNG-AS1*, *JRKL-AS1*, *KCNMB4*, *LOC100129940*, *LOC100507195*, *LOC100507250*, *LOC100507420*, *LYZ*, *MDM1*, *MDM2*, *MEF2C-AS1*, *MIR1279*, *MIR3913-1*, *MIR3913-2*, *MIR6074*, *PTPRB, RAP1B, SLC35E3*, *SNORA70G*, *STARD13-AS*, *TMEM161B-AS1*, *TRHDE-AS1*, *WHSC1L1,* and *YEATS4*.

### Correlation between amplifications of rPA and CXPA

When we compared rPA with CXPA at different stages of invasion, two amplifications were shared to rPA, mCXPA and fCXPA: *RPSAP52* and *HMGA2*. Among all the amplifications, *GRB7* and *ERBB2* amplifications were observed in rPA and iCXPA. Interestingly, 18 genes were shared to rPA and fCXPA: *CETN3*, *GPR98*, *KL*, *LINC00423*, *LINC01339*, *LOC100129940*, *LOC102546226*, *LOC731157*, *LYSMD3*, *MBLAC2*, *MEF2C*, *MEF2C-AS1*, *MIR3660*, *MIR9-2*, *POLR3G*, *STARD13*, *TMEM161B*, and *TMEM161B-AS1* (Fig. 2f).

**Figure 3:**
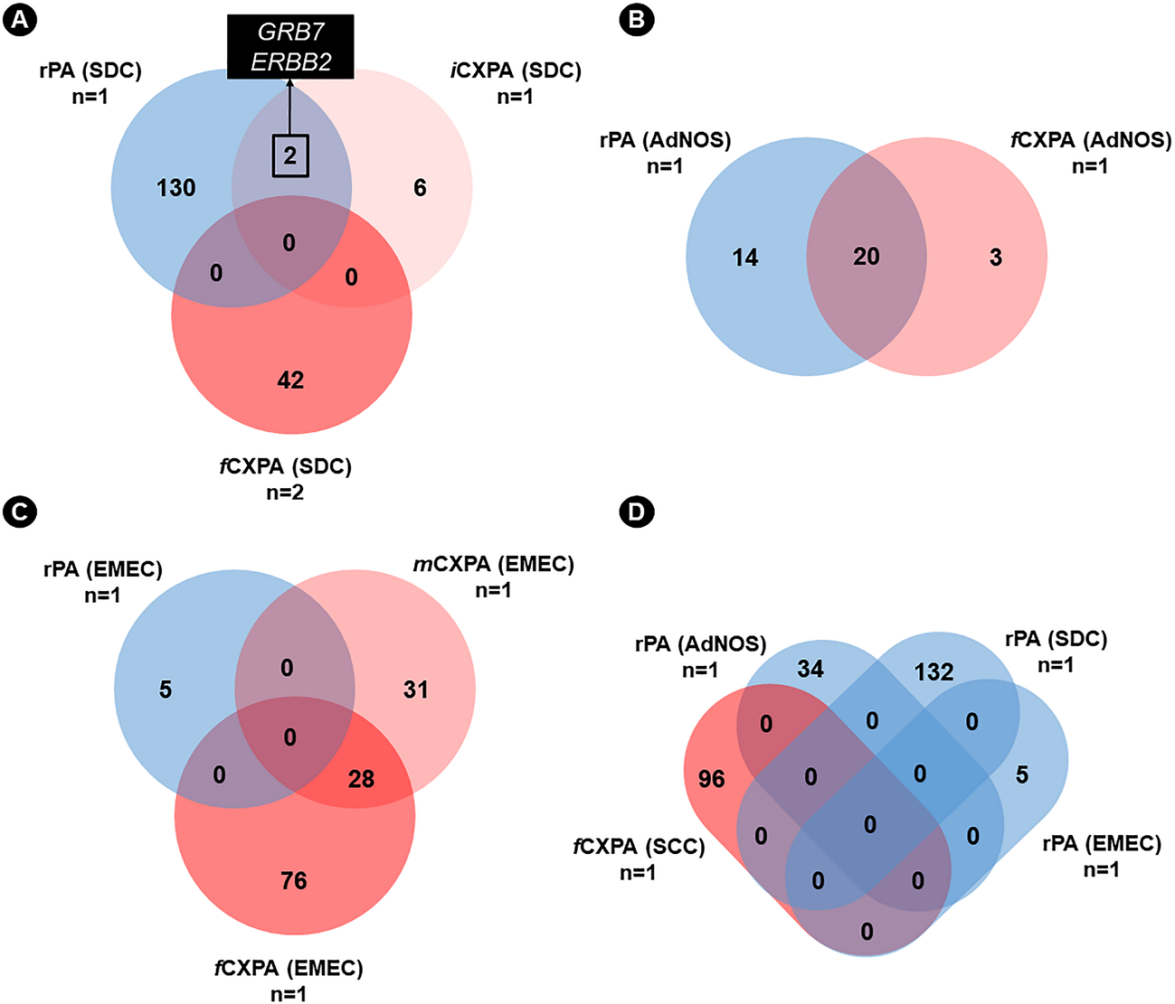
CNA profile in CXPA by histologic subtype. (**A**) CNA profile in CXPA (SDC subtype). The majority of amplifications in the benign residual area were unique, with amplifications in *GRB7* and *ERBB2* being common to both rPA and intracapsular (iCXPA). (**B**) CNA profile in CXPA (AdNOS subtype). Most of the amplified genes in the malignant area were shared with the residual area. (**C**) CNA profile in CXPA (EMEC subtype). None of the amplifications in the transformed areas were shared with the rPA. (**D**) CNA profile in CXPA (SCC subtype) compared to rPA subdivided by histologic subtype. All amplifications in CXPA (SCC subtype) were unique and did not share alterations with any of the rPAs analyzed. *CXPA* carcinoma ex pleomorphic adenoma, *rPA* residual pleomorphic adenoma, *AdNOS* adenocarcinoma NOS, *SCC* squamous cell carcinoma, *EMEC* epithelial–myoepithelial carcinoma, *MC* myoepithelial carcinoma, *SDC* salivary duct carcinoma, *SC* sarcomatoid carcinoma, *iCXPA* intracapsular CXPA, *mCXPA* minimally invasive CXPA, *fCXPA* frankly invasive CXPA, *n* number of samples included in the analysis.

In an attempt to observe whether the amplification of the malignant area in each histologic subtype was shared with the residual benign area, we compared the rPA alterations with CXPA separated by histologic subtype and degree of invasion. When comparing the amplifications of the benign residual area and malignant area of CXPA (SDC subtype), we observed that most of the amplifications of rPA were unique (130–71.4%). Six of them (3.3%) were exclusive to the iCXPA (SDC subtype): *CDC6*, *CDK12*, *HER2*, *IGFBP4, LASP1* and *MLLT6;* and two amplifications (1.1%) was shared by the rPA and the iCXPA (both SDC subtype): *GRB7* and *ERRB2* (Fig. 3a). When we compared the genes amplified in the rPA adjacent to the CXPA (AdNOS subtype) with the amplified genes of CXPA (AdNOS subtype) cases, we observed that 20 amplifications (54.1%) were shared and three (8.1%) were unique to the transformed areas: *LIC00461*, *PDS5B*, and *STARD13-AS1* (Fig. 3b).

There were no shared amplifications between benign residual areas and malignant areas of CXPA (EMEC subtype) (Fig. 3c). Finally, since we could not evaluate the bening residual area of CXPA (SCC subtype), we decided to compare the amplifications of malignant areas of this tumor with the rPA adjacent to other histologic subtypes that showed alteration. This analysis showed that all amplifications of the malignant area of the CXPA (SCC subtype) were unique (Fig. 3d).

### Gene ontology (GO) and pathway enrichment of amplified genes

To determine the most likely biological effects of the amplified genes, we performed gene ontology (GO) analysis for these aCGH data (Supplementary file 4). GO analysis revealed that CXPA presented with gene enrichment involving different biological processes and molecular functions. Among the top 10 enriched biological processes, five were directly related to the tumor immune response, including the *IFNA* and *MEF2* family genes in the processes of NK cell activation (*p* = 6.59E-16), lymphocyte proliferation (*p* = 3E-11), and T cell activation (*p* = 7.03E-11). Among the top 10 enriched molecular functions, the cytokine receptor binding (*p* = 1.89E-07) and cytokine activity (*p* = 4.94E-07) were identified as molecular functions related to the tumor immune microenvironment. Kyoto encyclopedia of genes and genomes (KEGG) pathway enrichment analysis was performed. Among the top ten enriched pathways, the RIG-I-like receptor signaling pathway (*p* = 6.53e-12), Jak-STAT signaling pathway (*p* = 8.29e-11), natural killer cell mediated cytotoxicity (*p* = 1.28e-08), Toll-like receptor signaling pathway (*p* = 1.36e-08), and NOD-like receptor signaling pathway (*p* = 1.36e-08) were highlighted. GO analysis of the genes amplified in rPA did not reveal any statistically significant biological processes or KEGG pathways. These genes were enriched solely in trace-amine receptor activity (*p* = 1.34e-05) (*TAAR2*, *TAAR5*, *TAAR6*, *TAAR8*, *TAAR9*), according to the molecular function analysis.

## Discussion

In this study, we used aCGH to evaluate residual benign and transformed areas in patients who developed CXPA in Brazilian individuals. Our findings represent the first study in the literature of multiple CNAs in CXPA, with some of these alterations also present in the adjacent benign tissue surrounding the transformation site. Crucially, our investigation highlights that the transition from PA to CXPA is characterized by a significant increase in the number of CNAs. This finding is supported by a previous and recent study from our group, which used aCGH to demonstrate an increase in the number of miRNA genes throughout the malignant transformation of PA^18^.

Our primary results revealed several key findings: (1) the chromosomal gain of *PLAG1* persisted throughout the malignant transformation, being present in both rPA and CXPA samples. (2) Amplification of *HMGA2* and *RPSAP52* was particularly prevalent in both groups analyzed. (3) *GRB7 and ERBB2* amplification may represent an early event in the malignant progression from rPA to CXPA (SDC subtype). (4) Genes showing amplification in the transformed regions of the tumor were found to be enriched for biological processes associated with immune signaling.

A gain indicates a higher copy number of a genomic region compared to a reference sample, suggesting duplication or a few additional copies of that region. On the other hand, the term amplification indicates a particularly pronounced and often substantial increase in copy number, typically representing multiple extra copies a given genomic segment. In our series, amplifications of *PLAG1* were not detected by aCGH; however, copy number gains of *PLAG1*, especially involving chromosomal region 8q, were observed in both residual benign areas and transformed areas. The pleomorphic adenoma gene 1 (*PLAG1*) produces a zinc finger protein with functions during embryogenesis and fetal development^19^. While specific alterations in *PLAG1* can be observed in lipoblastomas and other mixed skin and soft tissue tumors^20^, the role of *PLAG1* in salivary gland tumors is largely limited to PA and the CXPA^21^. Previous studies support chromosomal gain of *PLAG1* in the 8q12 region in PA^22^, and suggested that it may contribute to the malignant transformation of PA^13,19,23^. The involvement of rearrangements in this region in CXPA samples may further support the role of this gene in tumorigenesis^21^. Indeed, rearrangements in *PLAG1* have been identified as one of the most common genetic events in CXPA, regardless of histologic subtype^24^.

In a recent study of our group, we showed that the expression of the PLAG1 protein, and presumably its role, was maintained in the majority of rPA cases and in some CXPA cases^4^. However, data indicated that the expression of PLAG1 was reduced after transformation. Although we observed this loss of PLAG1 expression in malignant tissues, occasional discrepancies between PLAG1 protein expression and genetic status have been reported in the literature. Some investigators have suggested that a thorough investigation should be conducted to identify the underlying mechanism of this event in these tumors^11,25^.

Rearrangements in 12q14.3 involving the high mobility group AT-hook 2 (*HMGA2*), also known as *HMGI-C*, have been documented during the transformation of PA to CXPA^11,14^. In fact, abnormalities in *HMGA2* and *PLAG1* have been shown to be useful in distinguishing CXPA from de novo counterparts^24,26^, and the overexpression of these proteins may aid in the detection of CXPA, especially when a PA component is not evident^27^. *HMGA2* is recognized as a transcriptional co-regulator that is expressed at high levels during embryonic development, silenced in adult tissues, and re-expressed in several human cancers^28^. Previous observations have suggested that PAs with *HMGA2* amplification may carry an increased risk of malignant transformation^14^. Interestingly, in our study we show that although *HMGA2* amplifications were maintained in rPA, they were more prevalent in transformed areas.

In the correlation analyses between residual benign areas and transformed areas, we observed that, similar to *HMGA2*, amplification of the *RPSAP52* gene was prevalent in our CXPA samples. Surprisingly, *RPSAP52* is a transcribed RNA pseudogene that positively regulates *HMGA2* expression through the formation of an R-loop structure^29^. Under normal conditions, *RPSAP52* is also expressed in embryonic tissues and is silenced in most adult tissues^30^. In breast cancer and sarcoma, the *RPSAP52* pseudogene controls the *HMGA2/IGF2BP2/LIN28B* axis through positive transcriptional regulation of *HMGA2* and regulation of the function of the IGF2BP2 protein, which has pro-proliferative targets^30^. Thus, *RPSAP52* could be considered an oncogene whose dysregulation in CXPA could stimulate cell growth and maintain cells in a more undifferentiated state. This finding is unique in the literature, and to date there have been no reports of the presence of *RPSAP52*, especially in association with *HMGA2*, in rPA and CXPA. However, although it appears promising, methodologies that validate these findings should be applied in these tumors to understand the mechanisms that drive this process.

A comprehensive study analyzing 24 CXPA cases showed that of the 73 genomic alterations detected, 35.6% were amplifications, with *ERBB2* being particularly prevalent^31^. This finding is consistent with our study, which also demonstrated two amplifications of *ERBB2*: one in a case of iCXPA and another in a case of rPA. Amplifications in *CDK4*, *MDM2*, *ZNF703* and *FGFR1* were identified in both studies, further supporting their importance in CXPA pathogenesis.

Growth factor receptor-bound protein 7 (*GRB7*) is a multidomain adaptor protein co-opted by numerous tyrosine kinases involved in various cellular signaling pathways^32^. Its binding partner is the activated epidermal growth factor receptor (*EGFR*)^33^. The human *GRB7* gene is located on chromosome 17q12 and has been shown to be involved in the regulation of cell proliferation, migration and invasion in mammary, ovarian and esophageal tissues^34,35^. In our study, we showed that in CXPA (SDC subtype), *GRB7* and *ERBB2* genes are amplified in rPA and iCXPA. This finding suggests that these amplifications may be an initial step in the development of CXPA.

Furthermore, as a final highlight of this study, we observed that the amplified genes in CXPA were enriched for biological processes related to immune signaling. This discovery is particularly intriguing, because the amplified genes in rPA did not show a significant process and function profile in GO analysis. The study of the immune microenvironment in CXPA is limited, and this finding underscores the need to focus our attention on this crucial aspect of the carcinogenic process^36^.

Although the findings regarding *GRB7*, *ERBB2*, and *RPSAP52* amplification are significant, additional validations should be performed using different methods, such as fluorescence in situ hybridization (FISH) and/or immunohistochemistry (IHC), to further confirm and strengthen our results. This approach would allow a more comprehensive comparison and a deeper understanding of the results, especially when considering the analysis of genes such as *PLAG1* and *HMGA2*, which have been studied using these techniques. Therefore, considering that CXPA is a rare tumor arising from the accumulation of alterations in the pre-existing PA, we believe that our results highlight promising initial findings for further studies aimed at better understanding the malignant transformation of PA.

## Conclusion

In conclusion, this study is the first to explore CNAs in the malignant transformation of CXPA. The study’s results indicate that CXPA exhibited significant CNAs, some of which were also present in the adjacent residual benign area following transformation site. These CNAs included *PLAG1* gain, as well as amplification of *GRB7, ERBB2, HMGA2,* and *RPSAP52*. Furthermore, our study highlights a crucial aspect: the transformation process from PA to CXPA is associated with an increase in the number of CNAs. This underscores the stepwise development and progressive accumulation of alterations during the evolution of CXPA and sheds light on the dynamic nature of this transformation. These findings have the potential to inform more accurate diagnostic strategies and guide the development of personalized therapeutic approaches in the future. However, it is important to recognize that further studies are needed to validate these findings and translate them into tangible clinical applications.

## Material and methods

### Patients and tumor material

A total of 27 patients diagnosed with CXPA were included in this study. Formalin-fixed paraffin-embedded (FFPE) tissue sections of surgical specimens were used. The transformed area of all these cases and the benign residual area of 14 of them were analyzed. The benign residual areas of the other 13 cases of CXPA could not be analyzed by aCGH. Approval for all experimental protocols was obtained from the Institutional Ethics Committee of the Faculty of Medical Sciences of the University of Campinas (approval number: 2011/23366-5), ensuring compliance with the relevant guidelines and regulations throughout the study.

### Pathological analysis

The histological diagnosis of all cases was reviewed. Carcinomas were reclassified according to the extent of invasion beyond the PA capsule as (1) iCXPA (contained by the capsule); (2) mCXPA (infiltration of extracapsular tissue at a distance ≤ 1.5 mm) and fCXPA (infiltration > 1.5 mm). The histological analysis was evaluated according to the 2017 WHO histologic classification^37^.

### DNA extraction and genome-wide copy number analysis

Tumor DNA was extracted from a 1.5 mm-diameter puncture of FFPE using the Qiagen extraction kit (Qiagen GmbH, Hilden, Germany) according to the manufacturer’s recommendations and our previous studies^16–18^. To improve DNA quality, the protocol included dewaxing with xylene, followed by methanol washes and incubation in 1 mol/L sodium thiocyanate for 24 h. The tissue sediment was then dried and digested in high-dose proteinase K lysis buffer for 1.5 days. The material was purified on the column and eluted in the buffer. Samples of the tumor DNA and a reference DNA (collected from different healthy donors (Promega, Madison, WI, USA) were labeled with the Enzo genomic DNA labeling kit according to the manufacturer’s instructions. Five hundred nanograms of test DNA and 500 ng of reference DNA were co-hybridized with a 180 k oligonucleotide matrix (SurePrint G3 Human CGH Microarray 4 × 180 K design 22,060, Agilent Technologies, Palo Alto, CA, USA) according to Agilent procedures.

The genome-wide copy number analysis was realized according to Kimura et al. (2024)^18^. In summary, this work contained 24,011 exomic probes. Microarray images were acquired using the Agilent microarray scanner bundle, and data were extracted using feature extraction (v9.1) (Agilent Technologies, Santa Clara, CA, USA). aCGH data were analyzed using Nexus copy number discovery edition v7.0 software, according to Mariano et al.^17^. Genomic CNA was called based on the FASST2 segmentation algorithm (limit of significance defined in 5 × 10^−8^) with log2 limit ratios of 0.2 or 0.8 for high copy gains or losses, respectively, and − 0.2 or − 1.0 for homozygous losses or gains, respectively. A gain was considered when a region of DNA had one extra copy of genetic material compared to the reference genome. Amplification, on the other hand, was considered when a region of DNA was increased by multiple copies. A loss was considered when a region of DNA had fewer copies than the reference genome.

### Data analysis and statistical analysis

Correlation analysis of the amplified genes in the rPA and CXPA in different invasion phases and different histologic subtypes was performed using Venn diagrams^38^. In addition, the amplified genes identified in the study groups were subjected to gene ontology (GO) enrichment analysis^39,40^ and KEGG pathways^41–43^ using the STRING database^44^. The Mann–Whitney U Test was used to statistically evaluate the distribution of loss, gain, and amplification between rPA and CXPA. The Kruskal–Wallis test was used to analyze the distribution of amplification across histological subtype and degree of invasion. The statistical software SPSS (version 22.0) was used, and the significance level was set at 5%.

### Ethics declarations

This study adhered to the principles outlined in the Declaration of Helsinki. Approval for the study protocol was secured from the Institutional Ethics Committee of the Faculty of Medical Sciences of the University of Campinas (approval number: 2011/23366-5). Prior to participation, informed consent was obtained from all subjects involved in the study.

## Supplementary Information

Supplementary Table 1.

Supplementary Table 2.

Supplementary Table 3.

Supplementary Table 4.

## Data Availability

Data is provided within the manuscript or supplementary information files.
